# Supporting family care: a scoping app review

**DOI:** 10.1186/s12911-022-01906-6

**Published:** 2022-06-21

**Authors:** Katharina Bidenko, Sabine Bohnet-Joschko

**Affiliations:** Chair of Management and Innovation in Health Care, Witten/Herdecke University, Alfred-Herrhausen-Straße 50, 58448 Witten, Germany

**Keywords:** Informal caregiver, Mobile applications, mHealth, Need domains, Target groups

## Abstract

**Background:**

Mobile applications (apps) may provide family caregivers of people with chronic diseases and conditions with access to support and good information. However, thorough understanding of how these apps meet the main needs and requirements of the users is currently lacking. The aim of this study was to review the currently available apps for family caregivers and evaluate their relevance to main domains of caregiving activities, caregivers’ personal needs, and caregivers’ groups found in previous research on family caregivers.

**Methods:**

We conducted a scoping review on English-language and German-language apps for family caregivers on two major app stores: Google Play Store and iOS App Store. Apps were included if the main target group were family caregivers. Data were extracted from the app descriptions provided by the app producers in the app stores.

**Results:**

The majority of the apps was designed to assist caregivers in their caregiving activities. Apps were rarely tailored to specific groups of family caregivers such as young carers and their needs. Further, apps addressing caregivers’ personal health, financial security, and work issues were scarce. Commercial apps dominated the market, often intermediating paid services or available for users of specific hardware. Public and non-profit organizations provided best-rated and free-of-charge apps but had a very limited range of services with focus on caregivers’ health and training.

**Conclusions:**

Our results indicate that current apps for family caregivers do not distinguish specific groups of family caregivers, also they rarely address caregivers’ personal needs.

## Background

Due to increasing average life expectancy and decreasing average birth rates there is worldwide a growing proportion of people with chronic diseases and conditions in need of care, thus increasingly challenging health and care systems [1]. Family caregivers provide a bulk of long-term care [2]. However, caring for people with chronic diseases or conditions is often complex and results in high burden for caregivers [3]. Support services aim to provide family caregivers with vital information, skills, and support, but there is some evidence that caregivers might not be making use of them [4]. Some researchers suggest that the reason for the low use is that current support services are often hard to access, require high costs, and rarely meet caregivers’ needs [4, 5].

### Mobile apps for family caregivers

Mobile applications reveal a new area of support services as they empower family caregivers through better access to health care assistance and information. Family caregivers use apps to track the health status of the care recipient, learn more about caregiving, and communicate with other caregivers or health care providers [6]. There is an increased belief in the benefits of apps since they can improve self-management, skills, health, and quality of life of family caregivers [7–11].

The market of mobile applications is dynamic and complex, thus presenting a challenge for users to find appropriate solutions but also for app providers to identify unmet needs of their potential users. There are only a few reviews that described mobile applications for family caregivers and concluded that apps mainly address care recipients without sufficient regard to family caregivers [12, 13]. However, it remains unclear what needs and target groups of family caregivers are currently addressed by available apps. Understanding the ability of current mobile offers to meet the information and support needs of family caregivers is crucial if mobile solutions are to support informal care effectively and is therefore the topic of the current study.

### Family caregivers’ needs

Family caregivers’ needs have been widely investigated resulting in two overriding categories: caregiving activity support needs [14, 15] and caregivers’ personal support needs [16, 17].

Family caregivers usually perform a number of caregiving activities and report the need for information and support regarding those activities [18]. In the current study we consider five main caregiving activity domains presented in previous studies on family caregiving [15, 19, 20]: (1) personal care, (2) medical care, (3) household assistance, (4) support in organizational matters, and (5) supervision and social support. Personal care is the assistance with daily activities such as bathing, dressing, and nutrition and assistance with mobility [19]. Caregivers also provide medical care such as wound care, injections, and giving medicine [15]. Household assistance (shopping, laundry, or meal preparation) represents the third domain in our study [15]. Fourth, support in organizational matters denotes activities such as making doctor appointments, ordering medicine, or speaking with health professionals [20]. Finally, supervision and social support comprises looking after the care recipient, talking, walking, and doing joint leisure activities with them [15].

Providing informal care may affect caregivers’ professional, social and private life [21]. Therefore, it is important to consider not only the support needs of family caregivers in caregiving domains but also caregivers’ personal support needs related to their own health and life [16, 17]. Based on previous research [15–17] our study defines five domains of caregivers’ personal support needs: (1) maintaining one’s own physical and mental health, (2) social contacts and exchange of experiences, (3) work and care, (4) financial security, and (5) free-time opportunities and other activities.

Finally, previous research also showed that caregivers do not represent a homogeneous group [22]. They take on different caregiving responsibilities and face different care situations. A working daughter, who takes care of an elderly parent, has other needs than a retired husband taking care of his wife with dementia. Caregivers’ groups can be characterized by the care situation (e.g. time and duration of care) as well as caregiver’s sociodemographic factors (e.g. gender, age and employment). Caregivers in different groups have specific needs that should be considered when assessing support services.

### Objectives

The aim of this study was to review the currently available apps for family caregivers in terms of their relevance to (1) five main caregiving activity support needs, (2) five main caregivers’ personal support needs, and (3) different sociodemographic groups of caregivers and caregiving situations.

Our approach is unique in two ways. First, in contrast to previous app reviews we use a comprehensive set of caregivers’ need domains to give a more detailed insight into the types of support apps provide and identify need domains that are currently neglected. Second, we analyze which specific groups of family caregivers are addressed by the current app services and identify unnoticed segments of family caregivers. Segmentation of family caregivers as app users was not addressed by the previous research so far. Effective services and supports are based on the in-depth understanding of users’ perspectives. Family caregivers need specific information and support depending on their individual situation. Understanding their specific needs and different target groups is of primary importance to provide optimal support. Our study provides knowledge that helps to identify product gaps and develop need-based services for family caregivers in different caregiving situations. App providers and public authorities could consider the current findings to develop effective support apps oriented toward caregivers’ needs and communicate them to potential users’ in different target groups.

## Methods

### Overview

We applied a scoping review methodology based on Mun et al. to identify and characterize apps for family caregivers [23]. Our analysis is based on the information available for users in app stores. In February 2021, we conducted a search in Google Play for Android and in App Store for iOS. One author recorded the titles and the description of the apps and both authors assessed the sample for eligibility.

### Selection criteria

To identify apps addressing family caregivers it was decided to choose the following search terms: “caregiver”, “carer” and “caregiving”. We also included the same terms in German: “pflegende Angehörige” und “informelle Pflege”. Apps addressing specifically informal caregivers or both informal caregivers and the care recipient were included for further analysis. Apps developed for professional caregivers or caregiving organizations were excluded, since our study focuses solely on family caregiving. Duplicates and irrelevant apps were also excluded.

### Data analysis

We collected information based on the description in the app stores and inspected the websites for the apps that provided unclear store descriptions. The following information was extracted: apps store category, cost, provider name, number of downloads, rating, main purpose, main features, and user target groups.

We used a set of main caregivers’ need domains defined in previous studies [14–17, 19, 20] to analyze to what extent app providers consider their users’ needs and target groups when they introduce their apps in the app stores. One of the authors inspected the data and gave a binary code to each variable (1, if a given app provides support on a specific domain/ targets a specific group; 0, otherwise), the other author reviewed. Identified types of support and target groups are outlined in Table 2.

## Results

### Results of the search strategy

First apps in English were identified in Google Play Store and iOS App Store by searching the chosen terms: caregiver (367 results), caregiving (311 results), and carer (450 results). Then apps in German were identified by using search terms “pflegende Angehörige” (248 apps) and “informelle Pflege” (251 apps). A total number of 1627 apps resulted by the search terms. 771 duplicates were identified and excluded. 856 apps were screened to select relevant apps according to their titles and their description in the store. Apps addressing specifically informal caregivers or both informal caregivers and the care recipient were included for further analysis. 713 apps that did not meet the inclusion criteria were excluded. A total number of 143 relevant apps were selected for the current analysis.

### General features

Of the 143 apps identified by the search strategy, about a half (74 apps) were solely found in the Google Play Store, only eight apps originated solely from the iOS App Store, while the remaining 61 apps were found in both repositories. Almost half of the apps were in the category for health and fitness (46%), about a quarter (25.2%) were marketed for medical purposes, while the rest were divided across social networks (7.2%), education (6.5%), lifestyle (5%), and others (10.1%). The majority of the apps were offered free of charge (81.2%), 16.5% offered in-app purchases and only 2.3% had a download fee. The lowest price of the paid apps was 0.99 USD and the highest price was 199 USD for a timely unlimited premium account. The average price for in-app purchases or app downloads was slightly higher in iOS App Store (19 USD) than in Google Play Store (14 USD). There were much fewer ratings in the iOS App Store (8.3%) than in the Google Play Store (50.3%), which might be because the stores display rating information differently or since there are generally more users of Google Play Store than iOS App Store. On average, in both stores slightly over a half of users (55.8%) reported high ratings between 4 and 5 (the maximum score), about one third (31.2%) reported average ratings between 3 and 4, and 13% reported poor ratings between 1 and 3. The download numbers relate only to apps within the Google Play Store and show that caregiver apps are not very popular: A majority of the apps had a low number of downloads with 77.8% below 5.000 downloads and 97% below 100.000 downloads. The most downloaded app was an intermediary platform bringing together healthcare service providers and family caregivers searching for help with one million downloads and more. The largest portion of apps (86.7%) in both repositories were offered by private enterprises. Public authorities or institutions (health centers, universities, or hospitals) offered 4.9% of the apps, 5.6% of the apps were provided by non-profit organizations (caregivers associations or self-help groups), and 2.8% by private persons (Table 1).

**Table 1:** General features

General features	n (%)
*App store*	
Google Play	74 (51.7%)
iOS App store	8 (5.6%)
Both	61 (42.7%)
*App store category*	
Health and fitness	64 (46%)
Medicine	35 (25.2%)
Social networks	10 (7.2%)
Education	9 (6.5%)
Lifestyle	7 (5%)
Other	14 (10.1%)
*App costs*	
Free of charge	108 (81.2%)
In-app-purchases	22 (16.5%)
Pay per download	3 (2.3%)
Highest price (US-Dollar)	0.99 USD
Lowest price (US-Dollar)	199.0 USD
Average price (US-Dollar)	14 USD (Google Play); 199 USD (App Store)
*Rating*	
High (4–5)	43 (55.8%)
Middle (3–4)	24 (31.2%)
Low (1–3)	10 (13.0%)
*Downloads*	
up to 100	26 (19.3%)
100–500	28 (20.7%)
500–1000	17 (12.6%)
1000–5000	34 (25.2%)
5000–10.000	11 (8.1%)
10.000–50.000	13 (9.6%)
50.000–100.000	2 (1.5%)
100.000 and more	4 (2.9%)
*Provider*	
Private enterprise	124 (86.7%)
Public institution	7 (4.9%)
Non-profit organization	8 (5.6%)
Private person	4 (2.8%)

### Types of support

Almost all apps aimed at supporting caregivers in their caregiving activities on one or several domains; the number totaled to 131 accounting for 90% of all apps tested. About one-fifth of the sample were apps that support the arrangement of professional services (21%) in all five caregiving domains. Such apps represented service providers or market intermediaries that help family caregivers and providers to interact with each other. Further, apps offered information (25.2%), consultation (9.8%), and training (4.9%) on all five main caregiving domains. These apps provided tips, advice, guidance, and education on caregiving and delivered telemedical counselling. Further, apps offered support in organizational matters that include for example a calendar and organizer to coordinate caregivers’ activities, medication and appointment reminders, checklists and documentation tools (20.3%). Finally, about 36% of apps helped caregivers in providing supervision and social support for the care recipient. The common features were tracking health state and geolocation, fall detection, and instant personal access to the care recipient via e.g. video calls. Some apps in this category supported joint leisure activities between the caregiver and the care recipient.

Apps for supporting caregivers’ personal needs accounted for only 24.1% of the sample. Most of these apps promoted social contacts and experience exchange between caregivers (11.9%). Few apps (6.9%) helped caregivers to maintain their physical and mental health by providing information on self-care and psychological consultation. Five and a half percent of the apps addressed the need for financial security by providing information on available benefits and measures for financial support of informal care. Finally, apps offered support for working caregivers to allow them to better combine work and care responsibilities (4.1%). There were no apps found addressing the need for caregivers’ free time and leisure activities.

### Target groups

Almost half of the sample (46%) did not differentiate target groups of family caregivers. One of the largest target groups was caregivers of people with specific conditions (19.6%). The majority of these apps were aimed at supporting caregivers of people with dementia and Alzheimer’s disease. Further 18.9% of the apps addressed caregivers for elderly. Only 2.8% of the apps account for the sociodemographic characteristics of the caregiver with one app addressing young adult caregivers and three apps targeting employed caregivers. Some apps were available only for clients of care agencies or long-term care institutions (2.1%). About twelve percent of the apps were aimed at supporting users of specific hardware such as sensors, tablets, or cameras. Table 2 provides an overview of the types of support and target groups addressed by the apps in the sample.

**Table 2:** Types of support and target groups

Types of support	n (%)
*Support in caregiving domains*	
* All caregiving domains*	
Intermediaries and providers of services	30 (21%)
Information on caregiving	36 (25.2%)
Consultation on caregiving	14 (9.8%)
Training on caregiving	7 (4.9%)
Support in organizational matters	29 (20.3%)
Supervision and social support	52 (36.4%)
*Support for caregivers’ personal needs*	
Maintaining caregivers’ own health	10 (7%)
Social contacts and experience exchange	17 (11.9%)
Work and care	6 (4.2%)
Financial Security	6 (4.2%)
Target groups	n (%)
All caregivers	67 (46.9%)
Caregivers for elderly	27 (18.9%)
Caregivers of people with specific condition(s)	28 (19.6%)
Specific sociodemographic groups of caregivers (e.g. age)	4 (2.8%)
Clients of a health care or insurance company	3 (2.1%)
Users of specific hardware to support care	17 (11.9%)

### Types of support across ratings, providers, and payment modalities

**Fig. 1:**
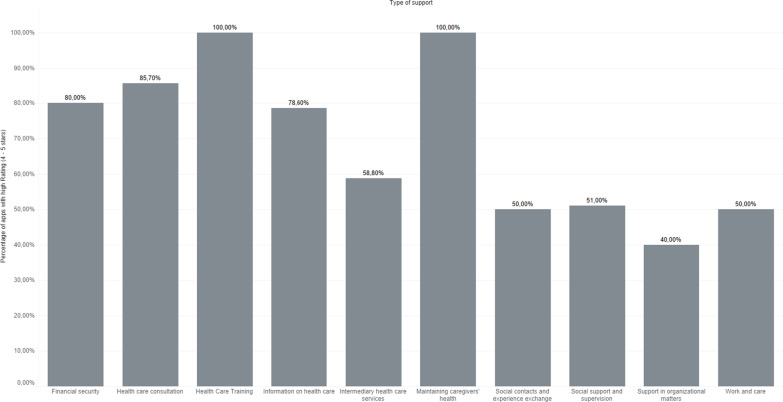
Chart comparing types of support across user rating

First, we focused on app ratings to provide an insight into caregivers’ opinion regarding different types of support. Reviewing the types of support according to the user rating (Fig. 1) revealed that more than 80% of the apps offering information, consultation, and training on caregiving had high ratings (4–5). Most positively rated apps offered training on caregiving (100% with ratings 4–5). Also, high ratings achieved apps that addressed caregivers’ health (100% with ratings 4–5) and financial security (80% with ratings 4–5). The lowest proportion of high ratings was among apps that provided support in organizational matters.

**Fig. 2:**
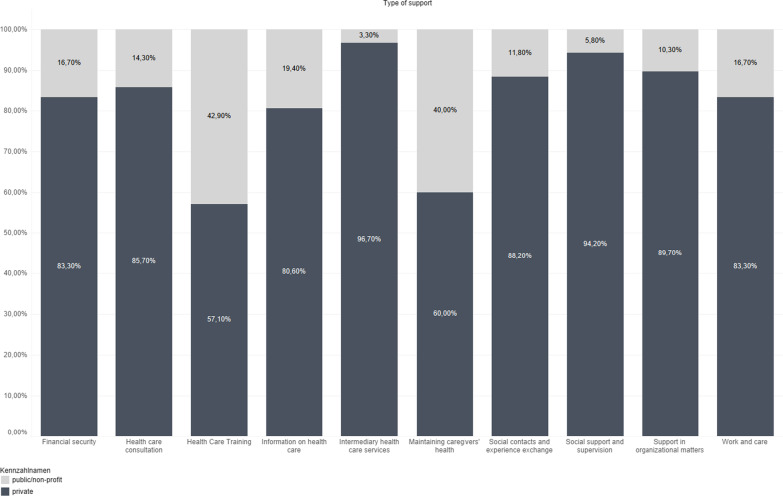
Chart comparing types of support across providers

Second, we analyzed app providers and their preferences regarding different types of services. Comparison of apps according to the app providers (Fig. 2) revealed that in almost all types of support provided by apps, private enterprises dominated over the public and non-profit organizations. Especially high was the number of private enterprises among intermediaries and providers of professional services as well as apps supporting supervision and social support: private providers accounted for more than 90% of the apps. In contrast, the best-rated types of support, training on caregiving domains, and support in maintaining caregivers’ own health were more often provided by public and non-profit organizations: private providers accounted for about 60% of the apps.

**Fig. 3:**
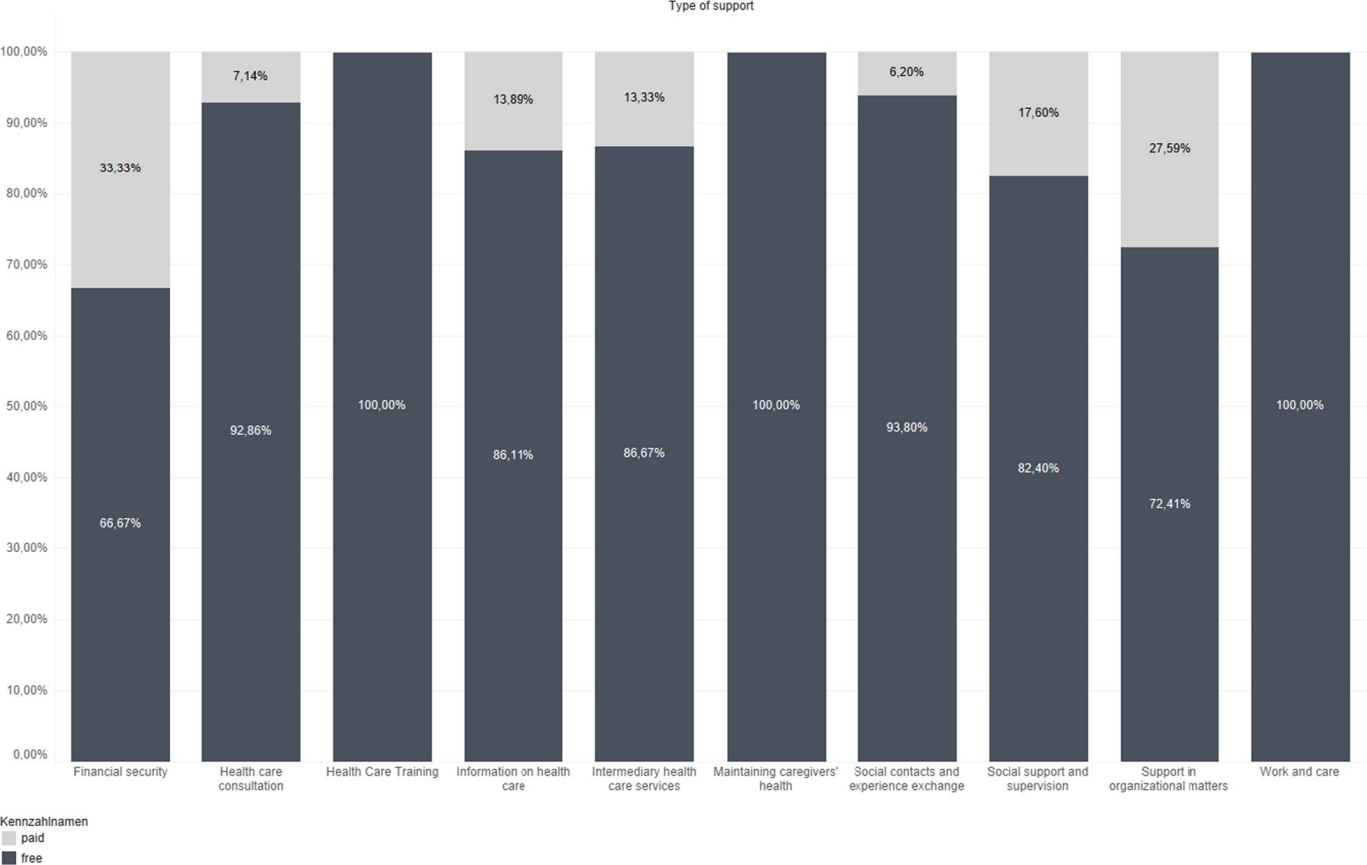
Chart comparing types of support across payment modalities

Third, we assessed payment modalities to give an insight into potential costs of different types of services. Figure 3 provides a breakdown of the support offerings of the apps according to whether apps were free or paid. The proportion of paid apps varied across types of support but accounted for not more than 33.3%. There were no payment requirements among apps offering training on caregiving and apps supporting caregivers in maintaining their own health. Low proportions of paid apps were among apps offering consultation on caregiving (7.1%) and among apps supporting social interaction and experience exchange between caregivers (5.9%). An especially high number of paid apps was in the category among apps providing support in organizational matters (27.6%). The highest number of paid apps was among apps supporting caregivers’ financial security (33.3%).

## Discussion

This study explored if and how mobile apps for family caregivers meet their needs. According to the previous research, apps are not sufficiently focused on the needs of family caregivers [13, 25]. We evaluated what needs and target groups of family caregivers are addressed by available apps. Our unique and comprehensive set of the ten main need domains provided an in-depth understanding of the app users’ perspective. Further, we addressed a group orientation of the apps, which received little attention so far, however, is crucial to provide need-based support for family caregivers. This study collected data from the two main app stores, Google Play Store and iOS App Store, and analyzed the content of 143 apps for family caregivers. We relied on a set of main domains of caregiving activity support needs and caregivers’ personal support needs derived from previous empirical research [15–17, 19, 20, 24]. Our results show discrepancies between the types of support provided by current services and caregivers’ needs that could be of high interest for app providers and policymakers. Based on our results, support services could be better adapted to the specific needs of family caregivers.

Overall, our analysis showed that apps mainly support caregivers’ needs in caregiving domains and are less targeted at caregivers' personal needs. This result supports previous findings indicating that apps are more focused on supporting caregivers in their caregiving role rather than caregivers’ own health as well as their private and professional life [13, 25]. In the following, we discuss in detail how identified apps match the main caregiving and personal need domains along with the target groups of family caregivers.

### Caregiving domains

The current apps offered extensive assistance in supervision and social support caregivers provide for the care recipient. Most of these apps were commercial and aimed at tracking care recipients’ health, state, or location on distance, which raises a number of privacy concerns regarding the use of collected personal data. Further, some apps required specific paid hardware like sensors or cameras even though the use of apps was mostly described as free of charge. Caregivers often experience financial burden caused by wage penalties and out-of-pocket costs [26] and would benefit from more affordable and accessible services.

Another widely addressed caregiving domain is the support in organizational matters. However, such apps contained mainly calendar, reminder, or documentation features and rarely addressed issues with caregiving arrangements and related bureaucracy. These apps were mostly commercial with a relatively high proportion of in-app purchases and relatively low ratings compared to other categories. Apps helping caregivers to identify eligible financial assistance and apply for it could be highly effective, since caregivers often find it difficult to interact with the health care system [27]. The affordability of such apps is important for caregivers in difficult financial situations.

Further many commercial apps were engaged in intermediation between family caregivers and providers of paid health care services. Such apps are distribution channels for various care agencies. Commercial background of these apps raises a question of caregivers’ access to complete and independent information about respite services. Direct technical assistance of caregivers in carrying out personal and medical care was not very common.

Especially high ratings received apps that offered information, training, and counseling on caregiving. There were more public and non-profit providers involved and fewer in-app purchases or other hidden costs for users. The best-rated category was training on caregiving with the highest proportion of public and non-profit providers and no paid apps. In total there were only few apps offering this type of support. In the future, public authorities that are responsible for providing support for family caregivers could pay more attention to apps as support measures.

### Personal need domains

As to the personal need domains, apps mainly addressed caregivers’ need for social contacts and experience exchange through social networks, forums, and support groups. There were only a few apps addressing caregivers’ own health. Caregiving may be burdensome leading to physical and psychological health problems among informal caregivers. Numerous studies suggest that family caregivers have poorer self-reported health compared to non-caregivers [28–30]. Caregivers report depression symptoms, pain, and increased drug consumption [29, 31]. Therefore, caregivers could profit from apps that help and motivate them to take care not only about the care recipient’s health but also of their own. More often public and non-profit organizations offered support in maintaining caregivers’ own health, and these few apps were more often highly rated by users.

Work and financial security are other important need domains since many caregivers have to combine work and caregiving, or even give up work completely, which leads to significant financial losses [32]. Compared to non-caregivers, caregivers are more likely to cut back schooling and working hours, quit jobs, and take unpaid time off of work [20, 33]. Such measures and out-of-pocket spending for care can erode financial security of family caregivers. Therefore, caregivers could profit from support on how to combine work and care or get access to financial and volunteer assistance. Our results show a clear lack of services to support the work and the financial security of caregivers. Family caregivers are especially at high risk of poverty as a result of high out-of-pocket costs of caregiving, reduced working hours or unemployment Further, it is important to notice that from the relatively small number of apps offering information and support in financial issues the proportion of paid apps was the highest. From a public health perspective, especially caregivers in difficult financial situations should be informed about available financial support without cost barriers. Finally, there were no apps found supporting the free time and leisure activities of caregivers.

### Target groups

Previous research results indicate that caregivers appreciate support targeted at their situation [34]. Family caregivers is a heterogeneous group with different needs and caregiving situations [22, 35]. Surprisingly, current applications are rarely tailored to specific groups of family caregivers, for example, young or working caregivers. An exception were a relatively high number of apps for family caregivers for people with specific diseases and conditions such as dementia. However, we were not able to find any apps addressing older caregivers, spouse caregivers, or men caregivers. These highly burdened groups of family caregivers could benefit from support services targeted to their specific needs [22].

Our results have shown that commercial providers currently have little focus on the needs of the caregivers. Public and non-profit organizations are trying to fill the gap by providing assistance aimed at the caregivers' own needs. However, these efforts are scarce, and the proportion and diversity of these applications is very small. In addition, we found that two best-rated types of support, training on caregiving and support in maintaining own health, are more often provided by public and non-profit organizations free of charge. Public agencies could lay more focus on the development of support apps that meet caregivers’ personal needs such as maintaining own health, balancing work and care and having enough financial resources.

## Limitations

Our research has also some limitations. First, due to the constant addition and removal of apps from the market, the result lists in the apps stores have a narrow window of validity. However, since we used a large sample of 143 apps, the addition or removal of single apps does not have a significant impact on our results in the short term. Second, the search was conducted in Germany. Replicating the results in another country might lead to different results. Therefore, we used two languages English and German for our search to cover international applications as well. Finally, our results are based on the information provided in the app description in the stores. If apps are to address informal caregivers effectively, it is of high importance that app providers consider their target users and speak to their needs when introducing their apps in the app stores. However, relying solely on the information available in the app stores cannot give a full picture of content and quality of the apps. Future research could provide a more detailed insight for a sample of apps for caregivers after downloading and testing features. The growing number of apps for family caregivers might allow future research to differentiate apps for certain stages of care or focus on specific groups such as caregivers of people with dementia or caregivers providing palliative care.

## Conclusions

In this paper, we reviewed mobile apps for family caregivers on two major app stores, Google Play Store and iOS App Store. We described the types of support provided by apps and analyzed their relevance to meet the main domains of caregivers’ needs. We showed that there are apps available with the potential to assist caregivers in their caregiving activities, while caregivers’ personal needs such as maintaining one’s own health, having free time or balancing work and care are being neglected. There were also no app services for groups of caregivers with specific needs. Based on our results app providers can expand their services and include apps oriented toward users’ needs.

## Data Availability

The datasets analysed during the current study are available from the corresponding author on reasonable request.
